# The Different Phytochemical Profiles of *Salvia officinalis* Dietary Supplements Labelled for Menopause Symptoms

**DOI:** 10.3390/molecules29010094

**Published:** 2023-12-22

**Authors:** Valentina Maggini, Gianpaolo Bertazza, Eugenia Gallo, Vittorio Mascherini, Lorenzo Calvi, Chiara Marra, Francesca Michelucci, Chiara Liberati, Anna Trassi, Rita Baraldi, Fabio Firenzuoli

**Affiliations:** 1 Research and Innovation Center in Phytotherapy and Integrated Medicine—CERFIT, Referring Center for Phytotherapy of Tuscany Region, Careggi University Hospital, 50134 Florence, Italy; eugenia.gallo@unifi.it (E.G.); mascheriniv@aou-careggi.toscana.it (V.M.); 2 Institute of Bioeconomy, National Research Council (IBE CNR), Via Gobetti 101, 40129 Bologna, Italy; gianpaolo.bertazza@ibe.cnr.it (G.B.); rita.baraldi@ibe.cnr.it (R.B.); 3 Independent Researcher, Via Fratelli Cervi 14, 27100 Pavia, Italy; lorenzocalvi@yahoo.it; 4 Casa Medica, Via Camozzi 77, 24121 Bergamo, Italy; chiara.marra@casamedica.it; 5 DAI Anesthesia and Rianimation, University Hospital of Pisa, Via Roma 67, 56126 Pisa, Italy; f.michelucci@ao-pisa.toscana.it; 6 Clinn srl, Piazza Vesuvio 19, 20144 Milan, Italy; c.liberati@clinn.it; 7 General Practioner ASL Central Tuscany, Piazza IV Novembre 28, 51035 Pistoia, Italy; annatrassi@gmail.com

**Keywords:** phytotherapy, extracts, *S. officinalis*, thujones, polyphenols, flavonoid

## Abstract

Phytochemical screening of four commercial products containing *Salvia officinalis* was carried out. Total phenolic content was estimated spectrophotometrically through the use of the Folin–Ciocalteau method, flavonoid content was measured through the use of aluminum chloride and 2,4-dinitrophenylhydrazine colorimetric assays, and isoflavones and α/β-thujones were analyzed through the use of high-performance liquid chromatograph (HPLC) and the gas chromatographic method. The analyses revealed the absence of thujones and isoflavones (i.e., genistin, genistein, and daidzein) in all four different extracts. The content of polyphenolic compounds varied among the samples, with the extract T being richer in both polyphenols and flavonoids than the other products by 1.8–3.2 and 1.4–4.0 times, respectively (*p*-value < 0.05). These results highlight the importance of quality control in salvia-based products since a thujone-free extract rich in polyphenols and flavonoids could be a good candidate for further preclinical and clinical studies to identify an effective herbal approach suitable for the long-term therapy of menopausal symptoms.

## 1. Introduction

During the menopause, quality of life can be affected by several complaints (e.g., hot flushes and impaired sexual function), with them lasting up to 15 years in 10% of women. The current treatment for menopausal symptoms is hormone therapy with estrogen and progesterone, which is associated with an increased risk of developing stroke, breast cancer, and thromboembolic, genitourinary, and pulmonary disorders [1]. Thus, there is dynamic research into therapeutic alternatives such as phytoestrogens, non-steroidal plant compounds acting on estrogen receptors [2], and other phenolic compounds with anti-inflammatory and antioxidant properties [3,4]. In traditional European medicine, *S. officinalis* is used as a bitter tonic for dyspepsia and gastrointestinal atony, a topical anti-inflammatory for skin and throat inflammation, and an expectorant for bronchial forms of excess mucus. The bioactive compounds of *Salvia*’s phytocomplex include mono- and di-terpenes such as 1,8-cineole, carnosic acid, carnosol, and ursolic acid [5]. *S. officinalis* also contains flavonoids (luteolin, apigenin, and quercetin glycosides) that are considered phytoestrogens and lignans that are non-flavonoid polyphenols. The human gut microbiota converts lignans into enterolactone and enterodiol, which play anti-inflammatory and antioxidant roles, with them acting as estrogen receptor modulators [6]. In fact, the anti-inflammatory, antioxidant, antiosteoporotic, antimicrobial, and antiviral properties of (poly)phenols and flavonoids have been extensively reported [7,8]. In addition, health benefits are shown in individuals with sarcopenia, cognitive decline, and metabolic and cardiovascular diseases [9,10,11,12].

*S. officinalis* is traditionally used to counteract excessive sweating, associated hot flushes, and physical and mental fatigue in the menopause [13,14,15]. In fact, the perimenopause and the menopause are accompanied by symptoms that can last from 6 months to more than 10 years with varying degrees of severity. Most perimenopausal women complain of demanding vasomotor symptoms such as hot flushes during the day and at night and predominantly night sweats. The etiology of hot flushes is unknown, and they seem to arise from altered thermoregulation in the hypothalamic region equipped with thermosensitive neurons [16]. Symptoms such as fatigue, cognitive changes, sleep disturbances, vaginal dryness, recurrent cystitis, osteoporosis, emotional lability, and even depression concur with the syndrome, forcing a high percentage of women to seek medical attention.

All of these symptoms are the consequence of the abrupt decrease in circulating hormones. Estrogens modulate central neural circuits, and the loss of a reliable estrogen rhythm weakens thermal homeostasis by demodulating serotoninergic and noradrenergic receptors (i.e., estrogens modulate the production, release, and reuptake of serotonin and norepinephrine and the activity of their receptors) [17]. In addition, at the level of the central nervous system, clinical trials have shown that *Salvia* spp. improves cognitive performance in patients with mild-to-moderate Alzheimer’s disease, with it interacting with the cholinergic system [18].

*S. officinalis* induced proliferative changes in the uteri of treated mice, suggesting estrogenic activity by some components of phenolic origin [19,20]. *S. officinalis*‘ hormonal effects have been investigated in vivo. The serum levels of follicle-stimulating and luteinizing hormones decreased in ovariectomized immature female rats treated with *S. officinalis* ethanolic extracts and its ferulic acid [6]. Sage tea (containing phytoestrogens) was effective in reducing the bone loss that occurred in aged female rats, probably by reducing bone turnover via the inhibition of bone resorption [21]. Due to the presence of substances with phyto-estrogenic activity, sage may affect implantation media because decidual angiogenesis has a main role in the maintenance of pregnancy at implantation time [22]. Other potential mechanisms of action of *S. officinalis* have been reported [16]. Clinical studies on the treatment of menopausal symptoms demonstrate that *S. officinalis* improves not only vasomotor symptoms but also exerts a positive impact on somato-vegetative and psychological symptoms, thus addressing a wide range of menopausal disorders [15,23]. The results of a double-blind study on a group of 80 menopausal women randomized to receive tablets of 3400 mg of ethanol extract of *S. officinalis* or a placebo for 4 weeks showed the effect of *S. officinalis* on the restoration of unbalanced brain waves [23]. A systematic review and meta-analysis reported the results of four clinical studies (310 patients) on the effect of *S. officinalis* on hot flushes in postmenopausal women (one study was conducted with the product M analyzed in the present work). Hot flush frequency significantly decreased in the treatment groups compared to the placebo group [24]. Although this effect was originally attributed to the presence of phyto-estrogenic bioactive compounds only [6], the role of the entire class of polyphenolic compounds in contributing to the different sage mechanisms of action has arisen [25]. Furthermore, new components with novel therapeutic properties are constantly being discovered [26].

According to the EU General Food Law Regulation (EC) No. 178/2002 [27], dietary supplements are considered foodstuffs and the business operator placing the product on the market has the responsibility for the safety of these products. Several guidelines provide information on the documentation and quality controls to be carried out for a safe production process and finished supplement. The characterization of the product’s chemical composition is not foreseen; hence, a lack of standardization between different supplements formulated with the same medicinal plant can arise. Therefore, the aim of our research is to analyze the chemical composition of four commercial products formulated with *S. officinalis*.

## 2. Results

### 2.1. Polyphenols and Flavonoids in S. officinalis Extracts

**Figure 1:**
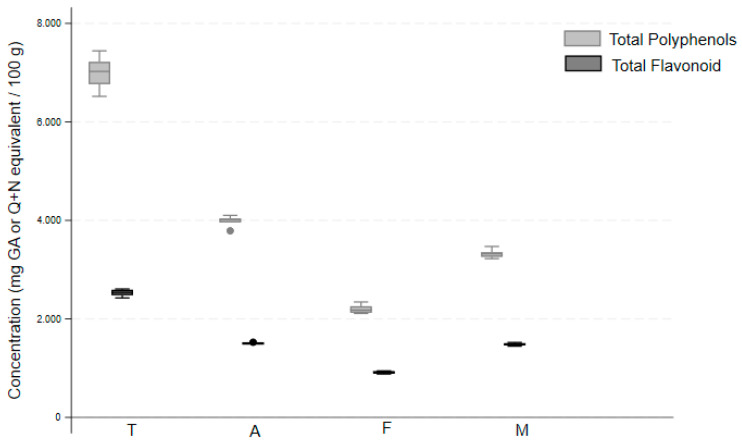
Comparison of total polyphenol and total flavonoid concentrations in four different *S. officinalis* extracts. Products: SALVITILAB^®^ (T), ACEF (A), Fontana (F), and Menosan^®^ (M). GA, gallic acid; Q, quercetin, N, naringenin.

Sage leaves from SALVITILAB^®^ were characterized by the highest content (median value, Figure 1) of total polyphenols (7024 mg/100 g) and flavonoids (2539 mg/100 g), almost twice the content compared to ACEF (4016 and 1503 mg/100 g, respectively) and Menosan^®^ (3323 and 1478 mg/100 g, respectively), while the lowest content was recorded for Fontana (2179 and 924 mg/100 g, respectively). These differences were statistically significant (Fisher’s exact test *p*-value < 0.0001), as confirmed by pairwise comparisons (Supplementary Tables S3 and S4).

### 2.2. Isoflavones in S. officinalis Extracts

**Figure 2:**
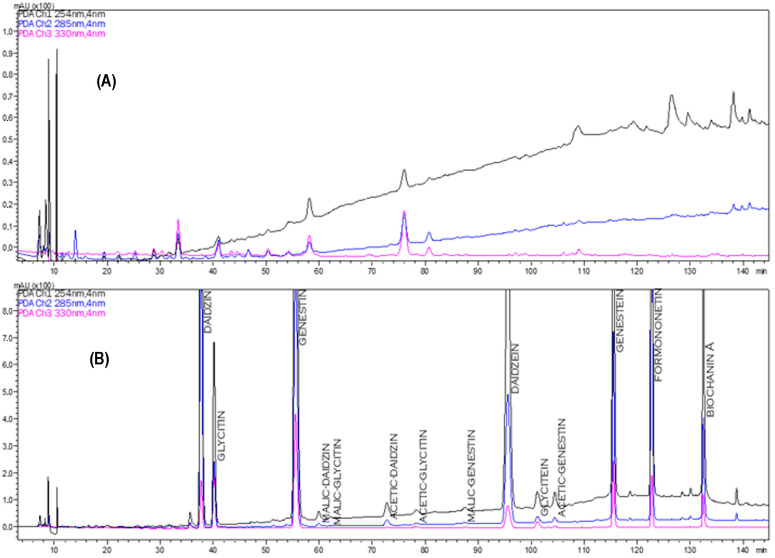
HPLC chromatogram of the *S. officinalis* extract of the SALVITILAB^®^ sample (**A**) and the isoflavones of soybean and red clover extract (**B**).

A representative HPLC chromatogram of isoflavones from an extract of SILVITILAB sample (A) as an example is shown in Figure 2 in comparison with typical isoflavones identified with the same chromatographic procedure in soybean and red clover extract (B).

Daidzin was the most abundant isoflavone recognized in all of the extracts, followed by genestin and genestein (Table 1). The ACEF extract contained the highest total isoflavones (1.521 mg/100 g), while the lowest isoflavones were determined in the SALVITILAB^®^ (0.1096 mg/100 g) and Menosan^®^ (0.0220 mg/100 g) samples, where genestin was below the LOQ. These differences were statistically significant as confirmed by pairwise comparisons (Supplementary Table S5).

### 2.3. Thujones in S. officinalis Extracts

**Figure 3:**
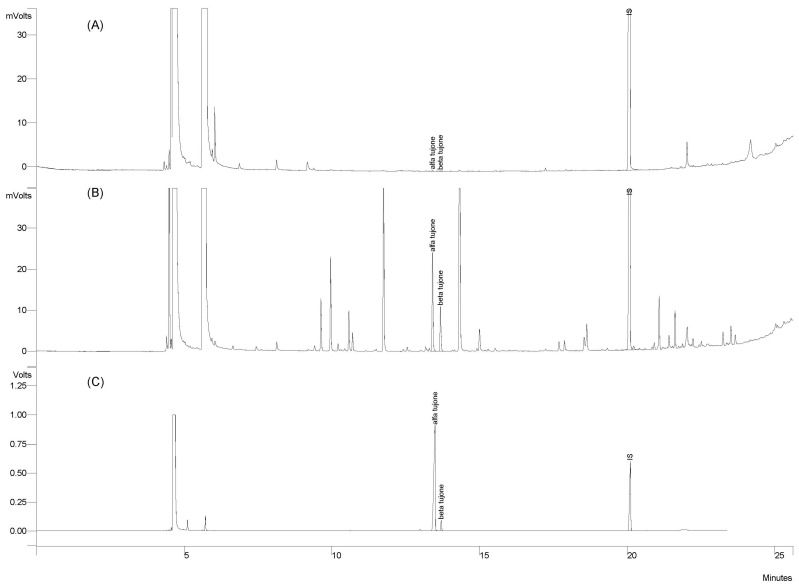
Representative GC chromatogram of the *S. officinalis* extracts SALVITILAB^®^ (**A**) and the control fresh sage (**B**) and standards (**C**) showing the peaks and retention time (Rt) of α-thujone (Rt: 13.49 min), β-thujone (Rt: 13.70 min), and the internal standard (IS) ethyl decanoate (Rt: 20.10 min).

Representative GC chromatograms of α- and β-thujones in the sage products are shown in Figure 3A, compared with the control fresh leaves (Figure 3B) and standards (Figure 3C) for identification validation.

The data reported in Table 2 show that β-thujone resulted below the LOQ in all samples as well as α-thujone in extract F. Extract M showed the highest value of α-thujone (0.001 mg/100 g), while extract T showed a significantly lower value than samples A and M (*p*-value < 0.0001) as also validated by Tukey–Kramer pairwise comparisons (Supplementary Table S4).

## 3. Materials and Methods

### 3.1. Materials

#### 3.1.1. Samples

The samples used for the investigation were four commercial products: Menosan^®^ tablets (M) (ethanolic extract of freshly harvested *S. officinalis* L. from Himalaya Wellness—Warszawa, Poland), SALVITILAB^®^ (T) (ethylene glycol extract of dried sage from Tilab srl—Erba, CO, Italy), *S. officinalis* dried extracts from Sergio Fontana srl (F) (Canosa di Puglia, BT, Italy), and ACEF (A) Spa (Fiorenzuola D’Arda, PC, Italy). The Menosan^®^ capsules were easily pulverized with a mortar and stored at room temperature. Powdery samples of freeze-dried glycolic extracts of sage from the Tilab food supplements producer and the Fontana pharmacy were stored at room temperature.

Furthermore, fresh sage leaves, grown in the Institute of Bioeconomy nursery, were used to check the validity of the analytical procedure and for retention time confirmation. The samples were minced with a pestle and mortar in the presence of liquid nitrogen. These samples were stored in a freezer at −20 °C until analysis.

#### 3.1.2. Chemical Products

The methanol, isopropanol, dioxane, and dichloromethane were purchased from VWR International (Radnor, Pennsylvania-US). The acetonitrile and ethanol were purchased from Carlo Erba, (Cornaredo-Milano, Italy). The aluminum chloride was purchased from Alfa Aesar (Haverhill, MA, USA). The natrium carbonate and 2,4-Dinitrophenylhydrazine (2,4-DNPH) were purchased from Fluka-Fisher scientific (Rodano-Milano, Italy). The potassium acetate and methanesulfonic acid were purchased from Acros Organics-Fisher scientific (Rodano-Milano, Italy). The thujone, daidzin, genistin, daidzein, genistein standards, and ethyl decanoate were purchased from Sigma-Aldrich-Merck (St. Louis, MO, USA).

#### 3.1.3. Instruments

The Spectrophotometer Ultraviolet-Visible V-530 was purchased from Jasco (Tokio, Japan). The HPLC Nexera2 is composed of the following units: an LC-30AD liquid chromatograph, an SIL-30AC autosampler, a CTO-20AC prominence column oven, an SPD-M30A diode array detector, and a DGU-20A5R degassing unit, purchased from Shimadzu (Kyoto, Japan). The gas chromatograph (GC) CP3800 was purchased from Varian (Palo Alto, CA, USA).

### 3.2. Phenols Analysis

#### 3.2.1. Extraction of Phenols

All samples were extracted with a methanol:water (50:50 *v*/*v*) solution [28]. This ratio was chosen to obtain the best extraction since the samples from the Tilab food supplements producer and the Fontana pharmacy showed poor dispersion with extracting solutions containing larger aliquots of methanol or ethanol. Approximately 100 mg of SALVITILAB^®^ samples, Fontana, ACEF, and Menosan^®^ and 200 mg of chopped fresh sage leaves were added with 2 mL of methanol–water extraction solution. The mixtures were vortexed for 1 min and left to stand for 3 h at room temperature. Then, the mixtures were vortexed for 1 min and centrifuged at 7000 rpm for 8 min. The supernatants were collected, and the residues were suspended with another 2 mL of extractive solution. The samples were re-extracted exactly as previously described, and a total of five extractions were performed with a final volume of 10 mL. The phenol extracts were stored at +4 °C until analysis.

#### 3.2.2. Determination of Total Phenolic Content through Use of the Folin–Ciocalteu Colorimetric Method

The total phenolic content in the extracts was determined as described by Molina-Cortès et al. [29] with minor modifications. Phenolic extracts (0.2 mL) were added to a solution of 1.8 mL of methanol: water (50:50 *v*/*v*) and 1 mL of Folin–Ciocalteu solution and then vortexed for 30 s. Subsequently, to these solutions, 3.5 mL of 7.5% (*w*/*v*) sodium carbonate solution and 3.5 mL of water were added and then vortexed for 30 s. The samples were incubated for 30 min at ambient temperature in the dark and then centrifuged at 5000 rpm for 6 min. The absorbance of the solution was measured at 730 nm against a reagent blank. A standard curve of gallic acid was used to calculate the total phenolic content in the extract, which was expressed as gallic acid equivalents.

#### 3.2.3. Determination of Total Flavonoid Content through use of the Aluminum Chloride Colorimetric Method

The total flavonoids were determined as described by Chang et al. [30] with minor modifications. Approximately 2 ml of phenolic extracts was added to a 0.5 mL solution of AlCl3 10% (*w*/*v*) and vortexed for 30 s. The samples were then added to a 0.5 mL solution of 1 M potassium acetate and vortexed for 30 s. The samples were incubated for 30 min at ambient temperature and then centrifuged at 5000 rpm for 5 min. The absorbance of the solutions was measured at 430 nm against a reagent blank, as reported by Spiridon et al. [31]. A standard curve of quercetin was used to calculate the total flavonoid content in the extract, which was expressed as quercetin equivalents.

#### 3.2.4. Determination of Total Flavonoid Content through Ise of the 2,4-DNPH Colorimetric Method

The total flavonoids were determined as described [32] with minor modifications. Approximately 2 ml of phenolic extract was added to 1 mL of water and 2 mL of 2,4-DNPH reagent (1 g of 2,4-DNPH dissolved in 2 mL of 96% sulfuric acid and diluted to 100 mL with methanol). The samples were vortexed for 30 s and then heated at 50 °C for 50 min. After cooling, 5 mL of potassium hydroxide 10% (*w*/*v*): methanol (30:70 *v*/*v*) solution was added to the samples, and they were vortexed for 30 s. The samples were incubated for 30 min at ambient temperature and then centrifuged at 5000 rpm for 5 min. Approximately 1 mL of the supernatant was diluted to 5 mL with methanol. The absorbance of the solution was measured at 495 nm against a reagent blank. A standard curve of naringenin was used to calculate the total flavonoid content in the extract, which was expressed as naringenin equivalents.

### 3.3. Isoflavone Analysis

#### 3.3.1. Extraction of Isoflavones

Approximately 100 mg of SALVITILAB^®^, Fontana, Menosan^®,^ and ACEF samples and 200 mg of chopped fresh sage leaves were added to 0.5 mL of 96.6% ethanol [33]. The mixtures were vortexed for 2 min, allowed to stand at 4 °C for 4 h, and vortexed again for another 2 min. The mixtures were then centrifuged for 8 min at 9000 rpm. The liquid phase was separated and stored while the solid residue was added again with 0.5 mL of ethanol for a second extraction under the same conditions as the first one. A total of four extractions were performed, and the total extract content of 2 mL was stored in a freezer at −20 °C until analysis.

#### 3.3.2. Determination of Isoflavones through Use of the HPLC Method

Approximately 600 µL of isoflavonic extract were centrifuged at 14,000 rpm for 6 min, and 10 µL of extract was injected into the HPLC. The samples were eluted on two columns connected in series: the first was a Kinetex C18 5 µm 250 × 4.6 mm column (Phenomenex, Torrance, CA, USA) and the second one was an Atlantis dC18 5 µm 250 × 4.6 mm column (Waters, Milford, MA, USA). The elution of the analytes was performed with 4 solutions: (A) water and methanesulfonic acid at pH 3; (B) acetonitrile 65%, dioxane 10%, water, and methanesulfonic acid pH 3 25%; (C) methanol 65%, dioxane 10%, water, and methanesulfonic acid pH 3 25%; and (D) methanol 85%, dioxane 5%, and isopropanol 10%. The elution steps and ratios of the solutions are shown in Supplementary Table S1. The mobile phase flow rate was 0.5 mL/minutes and the column oven temperature was 24 °C. The isoflavones were identified by comparing the retention time and ultraviolet–visible spectra of the reference standards (daidzin, genistin, daidzein, and genistein; Supplementary Table S2) with the characteristic peaks of soybean and red clover extracts (Figure 1). Quantification was performed using the calibration curves of the reference standards.

### 3.4. Thujone Analysis

#### 3.4.1. Extraction of Thujone

Thujone was extracted using the method described by Scmiderer et al. [34] with minor modifications. Approximately 100 mg of samples from the Tilab food supplements producer, Fontana Pharmacy, ACEF, and Menosan^®^ and 200 mg of chopped fresh sage leaves were added, with 900 µL of dichloromethane and 100 µL of dioxane containing 0.9 mg of ethyl decanoate as the internal standard. The mixture was vortexed for 2 min, left to stand at 4 °C for 4 h, and vortexed again for another 2 min. Then, the samples were centrifuged for 8 min at 9000 rpm. The liquid phase was separated and kept while the solid residue was added again with 1000 µL of dichloromethane for a second extraction. The sample was re-extracted under exactly the same conditions as the first extraction. The extracts were merged, reaching a total volume of 2000, and then stored in the freezer at −20 °C until analysis.

#### 3.4.2. Determination of Thujone through the Use of GC Method

Approximately 2 µL of extract was injected into a GC equipped with an HP-1 column 60 m × 0.25 mm film 0.25 µm (Agilent technologies, Santa Clara, CA, USA) [35]. The oven temperature was initially 70 °C for 2 min and rose to 220 °C with a rate of 10 °C min^−1^ and to 350 °C with a rate of 20 °C min^−1^. The temperatures of the injector and flame ionization detector were 340 °C and 350 °C, respectively. Thujon was identified by the retention time of the reference standard.

### 3.5. Statistical Analysis

The concentrations of the different active principles in the analyzed samples were expressed as the mean value and the related standard deviation (SD). For the statistical analysis, the concentration values of the limit of quantification (<LOQ; <0.1 mg/L) were excluded. Differences in the median and mean concentrations among the different products were tested using the Fisher’s exact test and one way ANOVA test, respectively. Statistical analysis was performed using the Software STATA version 18. Statistical significance was considered for a *p*-value < 0.05.

## 4. Discussion

Our results showed that the T extract was the richest in both total polyphenols and flavonoids compared to the other products by 1.8–3.2 and 1.4–4.0 times, respectively (*p*-value < 0.05). These differences are expected since there is evidence that extraction techniques and parameters influence the bioactive profile of sage preparations [36] and potentially the relative health effect [9]. It can be hypothesized that the high content of polyphenolic compounds significantly contributes to *S. officinalis*‘ total bioactivity [25]. Referring to sage’s role in the management of menopause discomfort, controversial results have been reported about the pharmacodynamic mechanism of action. In regard to the potential estrogenic activity, our data excluded a substantial contribution by the isoflavones genistein, genistin, and daidzin since they represented a very low percentage of the total amount of polyphenols in all products, unlike what was reported for other medicinal plants such as *Trifolium pratense* L. used to treat menopausal symptoms [2]. Flavonoids possibly acting as phytoestrogens on menopausal symptoms have been reported in *Cimicifuga racemosa* L., *Humulus lupulus* L., and *Trigonella foenum-graecum* L. [37]. On the other hand, dopamine receptors binding and decreased prolactin release as well as serotoninergic system involvement have been proposed for flavonoids and other phenolic compounds contained in *Vitex agnus-castus* L., commonly used in relieving menopausal symptoms [37].

Sage also contains bioactive compounds with phyto-estrogenic abilities. For example, luteolin-7-O-glucoside and ferulic acid have been identified as putative estrogenic polyphenols [6]. Moreover, the polyphenol class contains lignans showing estrogen agonism/antagonism effects in the prevention and treatment of menopausal symptoms [38]. According to the literature, *S. officinalis* contains other bioactive compounds that show a beneficial role in improving quality of life during the menopause [39]. A decreased risk of breast cancer among post-menopausal women is associated with flavonol intake [40]. In addition, neuroprotective effects and antithrombotic activity have been reported for flavonoids and polyphenols from various herbal sources [41,42]. Further clinical trials on these chemical classes are necessary to improve knowledge about their clinical effects [25].

In the present work, the amount of α/β-thujone was also investigated. For all commercial products, β-thujone was below the LOQ and α-thujone ranged from not detectable (product F) to 0.001 mg/100 g (product M), which is below 10 parts per million (10 mg/kg), the max level established by the European Parliament to be a “free-thujone” alcoholic product [43]. As for the safety profile, thujone is naturally found in *S. officinalis* plants, and its consumption is associated with the onset of neurotoxic adverse effects [44]. In fact, the community *S. officinalis* monograph established that the amount of thujone must be specified in a product and the daily exposure must be below 6.0 mg [45]. Also, in clinical trials, the bioactivity of product M was demonstrated despite thujone depletion in the tested product [15,23]. Due to these safety and efficacy aspects, free-thujone products should be preferred.

The contents of the compounds analyzed in the tested samples agree with several studies obtained with similar methodologies. In fact, low levels of thujones were also found in sage leaves [15], and similar contents of polyphenols were observed [46]. The content of isoflavones in our samples are slightly higher compared to other sage species [47].

Also referring to the chemical composition of commercial products, it is important to underline that a lack of product standardization, a fundamental quality requirement, has been reported. For example, the analysis of *Ginkgo biloba* [48] or fermented red rice extracts [49], widely available on the international market, has shown variability among tested products in terms of flavonoids, terpene derivatives, and ginkgolic acids or monacolins, respectively. This wide-ranging content of active ingredients indicates that products marketed for the same indication are not pharmaceutically bioequivalent.

To the best of our knowledge, this is the first report comparing commercial products formulated with *S. officinalis*, and the product variability shown in this study emphasizes the importance of quality controls as reported for other herbal-based remedies [48]. However, preclinical and clinical studies are necessary to compare the efficacy and safety of the analyzed products.

## 5. Conclusions

This phytochemical analysis has shown how the composition of commercial extracts is different and potentially correlated to different therapeutic effects, with research efforts deserving to be deepened. For example, in relation to the products analyzed in this work, the thujone-free T extract, rich in polyphenols and flavonoids, could be an effective herbal approach and also suitable for the long-term therapy of menopausal symptoms. The next fundamental step will be to conduct preclinical and clinical studies to determine the efficacy and safety of the phytocomplex. Further characterization of the polyphenols group, to better define the active constituents of the central nervous system, responsible for phyto-estrogenic and cardioprotective activities, is also endorsed.

## Figures and Tables

**Table 1:** Isoflavones (mg/100 g ± SD) in *S. officinalis* extracts. Data from four separate experiments each performed in triplicate. Differences were considered significant when *p* < 0.05. Products: SALVITILAB^®^ (T), ACEF (A), Fontana (F), and Menosan^®^ (M). LOQ, limit of quantification.

Isoflavones Mean Value ± SD mg/100 g	T	A	F	M	*F* Test Statistic (*p*-Value from One-Way ANOVA)
Genestin	0.0105 ± 0.0082	0.1832 ± 0.0424	0.0174 ± 0.0056	<LOQ	116.96 (<0.0000)
Genestein	0.0076 ± 0.0054	0.1224 ± 0.0245	0.0170 ± 0.0048	0.0024 ± 0.0003	132.79 (<0.0000)
Daidzin	0.0914 ± 0.0761	1.2157 ± 0.0163	0.1898 ± 0.0735	0.0220 ± 0.0039	472.74 (<0.0000)
Total	0.1096 ± 0.0897	1.521 ± 0.0383	0.2242 ± 0.0766	0.0245 ± 0.0041	549.44 (<0.0000)

**Table 2:** Thujones (mg/100 g ± SD) in *S. officinalis* extracts. Data from four separate experiments, each performed in triplicate. Differences were considered significant when *p* < 0.05. Products: SALVITILAB^®^ (T), ACEF (A), Fontana (F), and Menosan^®^ (M). LOQ, limit of quantification.

Thujones Mean Value ± SD mg/100 g	T	A	F	M	*F* Test Statistic (*p*-Value from One-Way ANOVA)
α-thujone	0.0002 ± 0.0001	0.0009 ± 0.0001	<LOQ	0.0010 ± 0.0005	22.66 (<0.0000)
β-thujone	<LOQ	<LOQ	<LOQ	<LOQ	-

## Data Availability

The data presented in this study are available in the Supplementary Material.

## Supplementary Materials

The following supporting information can be downloaded at: https://www.mdpi.com/article/10.3390/molecules29010094/s1. Table S1: Ratio (%) of the four eluent solutions used for the HPLC runs during isoflavones determination. During elution, components of the mobile phase are varied with linear gradients; Table S2: Analytical standards used in this work (SigmaAldrich); Table S3: Polyphenols and flavonoids (mg/100 g ± SD) in S. officinalis extracts. Data are means from four separate experiments, each performed in triplicate. Differences were considered significant when *p* < 0.05. Products from SALVITILAB (T), ACEF (A), Fontana (F), and Menosan (M); Table S4: Tukey–Kramer pairwise comparisons for polyphenols and flavonoids of S. officinalis extracts (post hoc analyses for one-way ANOVA). Superscripts * indicates statistically significant (*p*-value < 0.05) estimates. SALVITILAB (T), ACEF (A), Fontana (F), and Menosan (M); Table S5: Tukey–Kramer pairwise comparisons for α-thujone and isoflavones of Salvia officinalis extracts (post hoc analyses for one-way ANOVA). Superscripts * indicates statistically significant (*p*-value < 0.05) estimates. SALVITILAB (T), ACEF (A), Fontana (F), and Menosan (M). LOQ, limit of quantification.
