# Supplementary material for: The Different Phytochemical Profiles of Salvia officinalis Dietary Supplements Labelled for Menopause Symptoms
Source: Molecules. 2023 Dec 22;29(1):94. doi: 10.3390/molecules29010094 (PMC10779573; doi:10.3390/molecules29010094)
Supplement: Supplementary file 1 [file molecules-29-00094-s001.zip › molecules-2775338-supplementary.pdf]

Supplementary Materials

The Different Phytochemical Profiles of *Salvia officinalis* Dietary Supplements Labelled for Menopause Symptoms

Valentina Maggini 1,*, Gianpaolo Bertazza 2, Eugenia Gallo 1, Vittorio Mascherini 1, Lorenzo Calvi 3, Chiara Marra 4, Francesca Michelucci 5, Chiara Liberati 6, Anna Trassi 7, Rita Baraldi 2 and Fabio Firenzuoli 1,*

1 Research and Innovation Center in Phytotherapy and Integrated Medicine—CERFIT, Referring Center for Phytotherapy of Tuscany Region, Careggi University Hospital, 50134 Florence, Italy
2 Institute of Bioeconomy, National Research Council (IBE CNR), Via Gobetti 101, 40129 Bologna, Italy
3 Independent Researcher, Via Fratelli Cervi 14, 27100 Pavia, Italy
4 Casa Medica, Via Camozzi 77, 24121 Bergamo, Italy
5 DAI Anesthesia and Rianimation, University Hospital of Pisa, Via Roma 67, 56126 Pisa, Italy
6 Clinn srl, Piazza Vesuvio 19, 20144 Milan, Italy
7 General Practioner ASL Central Tuscany, Piazza IV Novembre 28, 51035 Pistoia, Italy
* Correspondence: valentina.maggini@unifi.it (V.M.); fabio.firenzuoli@unifi.it (F.F.)

Table S1. Ratio (%) of the four eluent solutions used for HPLC runs in the isoflavones determination. During the elution, components of the mobile phase are varied with linear gradients.

Minutes	Ratio of eluent solutions (%)			
	A	B	C	D
0	80	20	0	0
5	80	20	0	0
29	72	28	0	0
32	70	28	2	0
56	54	20	26	0
78	40	6	54	0
87	34	0	62	4
96	28	0	62	10
141	0	0	48	52
147	0	0	20	80
150	0	0	0	100

Table S2. Analytical standards used in this work (SigmaAldrich)

**Compound**	**CAS number**
Thujone standard mixture	76231-76-0
Internal standard thujone: Ethyl deconoate	110-38-3
daidzin	552-66-9
genistin	529-59-9
daidzein	552-66-9

Compound	CAS number
genistein	446-72-0

Table S3. Polyphenols and flavonoids (mg/100 g ± SD) in *S. officinalis extracts*. Data are means from four separate experiments, each performed in triplicate. Differences were considered significant when $p < 0.05$. Products from SALVITILAB (T), ACEF (A), Fontana (F), Menosan (M).

Mean value ± SD mg / 100 g	T	A	F	M	F test statistic (p value from one way ANOVA)
**Total Polyphenols**	**6986 ± 318**	**3980 ± 119**	**2203 ± 98**	**3326 ± 96**	**640.24 (<0.0000)**
Flavonoids AlCl3	1274 ± 124	780 ± 5	316 ± 8	929 ± 8	160.48 (<0.0000)
Flavonoids DNPH	1257 ± 185	725 ± 13	598 ± 23	555 ± 28	54.47 (<0.0000)
**Total Flavonoids**	**2532 ± 66**	**1505 ± 12**	**914 ± 28**	**1483 ± 31**	**1531.35 (<0.0000)**

Table S4. Tukey-Kramer pairwise comparisons for polyphenols and flavonoids of *S. officinalis* extracts (post-hoc analyses for one-way ANOVA). Superscripts * indicates statistically significant (p value < 0.05) estimates. SALVITILAB (T), ACEF (A), Fontana (F), Menosan (M)

	T vs A	T vs F	T vs M	A vs F	A vs M	F vs M
	Total Polyphenols					
Mean difference	3006	4783	3660	1777	654	1123
TK-test	34.8134*	55.3954*	42.3876*	17.8245	6.5594	11.261
	Flavonoid AlCl3					
Mean difference	495	959	346	464	149	612
TK-test	15.7177*	30.4583*	10.9963*	12.7657*	4.089	16.8546
	Flavonoid DNPH					
Mean difference	532	659	702	127	170	43
TK-test	11.1717*	13.8517*	14.7513*	2.3210	3.1000	0.7791
	Total Flavonoid					
Mean difference	1026	1618	1048	591	22	570
TK-test	56.2039*	88.5915*	57.3974*	28.0485*	1.0336	27.0149*

Table S5. Tukey-Kramer pairwise comparisons for α-thujone and isoflavones of *Salvia officinalis* extracts (post-hoc analyses for one-way ANOVA). Superscripts * indicates statistically significant (p value < 0.05) estimates. SALVITILAB (T), ACEF (A), Fontana (F), Menosan (M). LOQ, limit of quantification.

	T vs A	T vs F	T vs M	A vs F	A vs M	F vs M
	α-thujone					
Mean difference	0.0007	C < LOQ	0.0007	C < LOQ	0.0000	C < LOQ
TK-test	7.5548*		7.9804*		0.3686	
	Genestin					
Mean difference	0.1727	0.0069	D < LOQ	0.1658	D < LOQ	D < LOQ
TK-test	20.6517*	0.8205		17.1743*		
	Genestein					
Mean difference	0.1148	0.0094	0.0052	0.1054	0.1200	0.0146
TK-test	25.8646*	2.1075	1.1743	20.5743*	23.4164*	2.8421
	Daidzin					
Mean difference	1.1243	0.0984	0.0694	1.0259	1.1937	0.1678
TK-test	48.5865*	4.2503*	2.9996	38.3963*	44.649*	6.2786*
	Total isoflavones					
Mean difference	1.4117	0.1146	0.0852	1.2971	1.4968	0.1997
TK-test	52.3545*	4.2487*	3.1583	41.6609*	48.0755*	6.4146*
